# Beauty’s Betrayal: *Mycobacterium abscessus* Case Series Following Aesthetic Procedures in the Brazilian Amazon

**DOI:** 10.3390/idr16040055

**Published:** 2024-08-07

**Authors:** Roberto C. C. Carbonell, Letícia L. F. Oliveira, Luis E. B. Galan, Eloise T. M. Filardi, Alysson B. M. Lins, Jânio J. M. Nattrodt, Domingos S. M. Dantas, Adilson C. A. Bernardi, Felipe A. Cerni, Manuela B. Pucca

**Affiliations:** 1 Medical School, Federal University of Roraima, Boa Vista 69310-000, Roraima, Brazil; rcccarbonell@yahoo.es (R.C.C.C.); luisbermejog@hotmail.com (L.E.B.G.); alyssonlins@hotmail.com (A.B.M.L.); felipe_cerni@hotmail.com (F.A.C.); 2 General Hospital of Roraima, Boa Vista 69305-455, Roraima, Brazil; janio_junior1@hotmail.com; 3 Graduate Program in Bioscience and Biotechnology Applied to Pharmacy, School of Pharmaceutical Sciences, São Paulo State University (UNESP), Campus Araraquara, Araraquara 14800-903, São Paulo, Brazil; e.filardi@unesp.br; 4 Programa Doutoral de Bioética da Faculdade de Medicina do Porto, 4050-290 Cidade do Porto, Portugal; saviojuazeiro@yahoo.com.br; 5 Department of Biology and Health Sciences, University of Araraquara (UNIARA), Araraquara 14801-320, São Paulo, Brazil; acabernardi@gmail.com; 6 Graduate Program in Tropical Medicine (PPGMT), State University of Amazonas, Manaus 69050-010, Amazonas, Brazil; 7 Department of Clinical Analysis, School of Pharmaceutical Sciences, São Paulo State University (UNESP), Campus Araraquara, Araraquara 14800-903, São Paulo, Brazil

**Keywords:** aesthetic procedures, non-tuberculous Mycobacteria, infections, cosmetic, mesotherapy, enzyme application

## Abstract

The Brazilian market holds the second position globally in the beauty sector, poised to surpass the USD 50 billion mark in the upcoming years. Aesthetic procedures encompass a spectrum, ranging from non-invasive ones, such as drainage, radiofrequency, ultrasound, and cryolipolysis, to more invasive techniques, including fillers, botulinum toxin, microneedling, micropigmentation, carboxytherapy, and enzyme application. This wide array of treatments has yielded satisfactory cosmetic results for individuals who opt out of surgical procedures. However, despite being categorized as having low complexity, they still carry inherent risks. These risks are often exacerbated by the breach of the skin barrier, the exposure of organs and spaces, or the presence of implantable devices. Among the bacteria most isolated concerning this matter are non-tuberculous Mycobacteria. This study presents descriptive case reports involving three patients under the care of the Infectious Diseases Department at General Hospital of Roraima (HGR). These patients were diagnosed with *Mycobacterium abscessus* infections subsequent to undergoing enzyme application procedures. Although these cases involve the same microorganism, they exhibit varying degrees of severity, ranging from the development of locally nodular formations to a progression towards sepsis. These cases provide an opportunity to delve into the diagnostic subtleties and clinical implications of these infections while also prompting a critical evaluation of therapeutic strategies. Additionally, the report underscores the potential risks associated with routine aesthetic procedures.

## 1. Introduction

The Brazilian and global markets are witnessing unparalleled growth in the beauty and aesthetics industry [1]. According to the International Society of Aesthetic Plastic Surgery, Brazil holds the second position worldwide for performing aesthetic surgical procedures, trailing only the United States [2]. With the industry’s remarkable advancement over the years, discussions and studies on managing its complications have gained increasing prominence. Among these, infections at puncture sites and surgical wounds caused by non-tuberculous Mycobacteria (NTMs) have drawn significant attention within the medical community [3].

NTMs can be categorized into two groups: (1) The first classification is based on their growth rate: rapidly growing Mycobacteria (RGMs), forming colonies on solid media within seven days, and slowly growing Mycobacteria (SGMs), taking over seven days to develop colonies on solid media. Typically, pulmonary and lymph node infections are associated with SGMs, while skin, soft tissue, bone, and joint infections are linked with RGMs. Moreover, these groups differ in terms of their susceptibility to antimicrobials. (2) The second classification is based on their pigment-producing ability: photochromogens produce pigments only in the presence of light, scotochromogens produce pigments under both light and dark conditions, and non-pigmented organisms do not produce any pigments [4].

NTMs are omnipresent, initiating growth within an hour and developing visible colonies within two to five days. They are found in natural water sources, water supply systems, soil, protozoa, animals, and humans, with numerous identified species. These microorganisms were formerly known as environmental Mycobacteria, later labeled “atypical” Mycobacteria—NTMs—or rapidly growing Mycobacteria—RGMs [5]. Notable RGMs include *Mycobacterium fortuitum*, *M. thermoresistible*, *M. neoaurum*, and *M. abscessus* [6]. Infections caused by these microorganisms are more prevalent in the skin and subcutaneous tissues and are typically associated with cosmetic surgical procedures such as liposuction; injectable solutions like procaine, L-carnitine, vitamin C, and lidocaine; silicone injections into breast prostheses; acupuncture treatments; nipple piercing placement; and skin infections after tattoos [5].

In Brazil, enzyme therapy procedures, popularly known as “fat melting”, are becoming very common in the cosmetic industry. These enzyme cocktails, sold by different companies, promise minimal pain and significant results in local fat reduction and body contour strengthening. Adipolytic therapy involves the subcutaneous injection of an enzyme solution several times into the relevant area by using a thin needle. The area is cooled with ice or treated with an anesthetic cream before the procedure. It is important to note that mesotherapy is a different technique from adipolytic therapy. Mesotherapy is an injection technique with medical and cosmetic applications and is often confused with injectable fat loss therapies. Mesotherapy describes non-ablative fat reduction using agents, such as beta-agonists, which activate adipocyte lipolytic pathways. In contrast, adipolytic therapy with biological detergents (e.g., deoxycholate) leads to permanent adipocyte ablation [7]. 

Three species that are clinically relevant and associated with infections from surgeries and cosmetic procedures are *M. abscessus*, *M. chelonae*, and *M. fortuitum* [8].

*M. abscessus* comprises a group of aerobic, non-encapsulated, non-spore-forming, and non-motile bacilli. They possess high lipid content in the cell wall region, increasing permeability to water, laboratory dyes, and disinfectants [9]. The precise transmission route of *M. abscessus* remains unknown. Nevertheless, certain similarities with *M. tuberculosis* are apparent [10]. The clinical presentation is usually nonspecific, characterized solely by local inflammatory signs without systemic manifestations (considered rare) [6]. 

Histopathological analysis reveals a granulomatous inflammatory infiltrate consisting of lymphocytes, histiocytes, epithelioid cells, and Langerhans giant cells [11]. Upon suspicion, drainage material undergoes microbiological analysis using the Ziehl–Neelsen method, and seeding can be performed in thioglycolate, blood agar, chocolate agar, MacConkey, and Lowenstein–Jensen media [12]. The appropriate treatment is determined based on the results of the antibiogram examination [6]. NTMs are notorious for their numerous mechanisms of antimicrobial resistance, such as low bacterial envelope permeability, drug export systems, drug-modifying enzymes, and biofilm formation ability [3]. The precise incidence of these infections following aesthetic procedures remains uncertain due to underreported complications. The most extensive population study on this subject showed an incidence of 2 per 100,000 individuals and nearly a threefold increase in NTM skin infections in the US over 30 years [13]. In Latin America, limited data are available concerning NTMs affecting the skin and soft tissues, primarily focusing on isolating these Mycobacteria in respiratory tract infections [14].

This study explores the complex scenario faced by patients diagnosed with *M. abscessus* infection after undergoing enzyme application procedures. Given the complexity and the limited available literature on this matter, there is an urgent call for a deeper understanding of its epidemiological and clinical aspects. This comprehension is pivotal to providing essential data for evaluating management approaches and gauging outcomes.

## 2. Case Description

This study was approved by the Research Ethics Committee of Federal University of Roraima, under protocol number CAAE 68910523.3.0000.5302 (approved on 2 June 2023), and written informed consent for publication from the patients was obtained after the three cases had been admitted and diagnosed. 

### 2.1. Case 1

The first case was a 28-year-old Caucasian female, single, medical student who used to engage in weightlifting five times a week, with no history of prior illnesses, comorbidities, or habits of alcohol or tobacco use. There were no reports of prior medication use or medication allergies and vaccines up to date, and she sought medical care at an infectious disease clinic in November 2021. She presented with painful reddish nodules on her flanks and lower abdominal area that had persisted for 4 months. The patient disclosed having undergone a lipolytic enzyme application procedure in Boa Vista, Roraima, Brazil, at the end of July 2021, aiming to reduce localized abdominal fat.

The lesions began appearing 2 months after the procedure—in September 2021—when she sought care from an infectious disease specialist in Rio de Janeiro, her place of residence. At that time, she underwent several examinations, including normal laboratory tests, a soft tissue ultrasound revealing Panniculitis, and collection puncture for culture, which was ultimately not performed. She was initially prescribed Cephalexin, 500 mg every 6 h for 15 days, but her condition worsened. In October 2021 (one month later), she was prescribed an empirical treatment regimen of Clarithromycin at 500 mg every 12 h, Levofloxacin at 750 mg once daily, and Rifampicin at 600 mg daily for four weeks, which did not yield improvement.

Returning to Boa Vista in November 2021—4 months post-procedure—she sought further care from an infectious disease specialist at General Hospital of Roraima (HGR), who conducted a lesion puncture and collected material for culture. During that period, the antibiotic regimen was altered, maintaining Moxifloxacin at 400 mg/day and Clarithromycin at 500 mg. On November 25th, the culture results indicated a positive finding for a non-tuberculous Mycobacterium in the 1st sample, while the 2nd sample tested negative. Bacilloscopy revealed 8 bacilli/100 fields in the 1st sample and 2 bacilli in 100 fields in the 2nd sample. Sensitivity testing demonstrated resistance to Streptomycin, Isoniazid, Rifampicin, and Ethambutol. The material was then sent to the Oswaldo Cruz Foundation for identification of the mycobacterial species and subspecies. The results, available in March 2022—4 months later—through the line probe assay (LPA), identified the species as *Mycobacterium abscessus* subspecies *bolletti*. This revealed sensitivity to Amikacin and Cefoxitin; resistance to Ciprofloxacin, Clarithromycin, and Tobramycin; and intermediate sensitivity to Doxycycline and Moxifloxacin.

**Figure 1:**
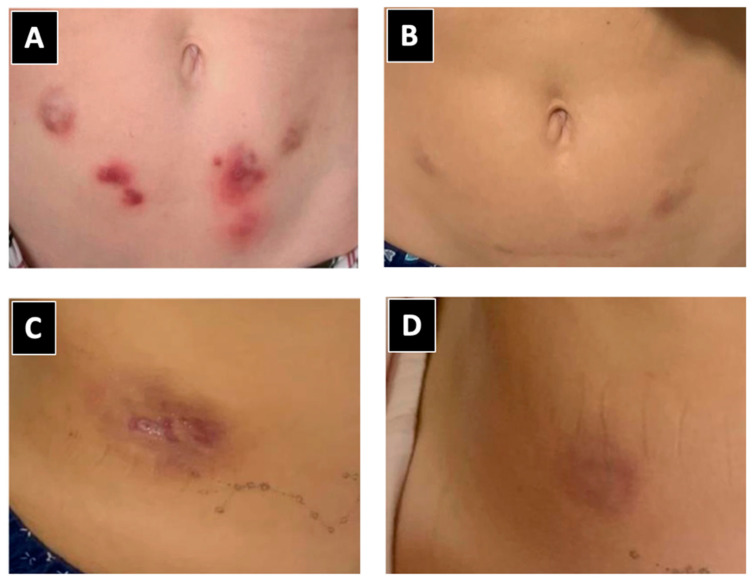
Characteristics of lesions caused by *Mycobacterium abscessus* post-lipolytic enzyme application. (**A**,**C**) Lesions and nodules identified prior to diagnosis. (**B**,**D**) Improvement following targeted treatment based on sensitivity testing.

Following the identification and sensitivity test results, Clofazimine at 100 mg/day and intravenous Amikacin at 500 mg/day for 15 days, followed by three times a week for the initial 3 months, were added to the treatment regimen of Clarithromycin at 500 mg every 12 h and Moxifloxacin at 400 mg once daily. Since then, there has been an improvement in the nodules (Figure 1). She is currently receiving treatment in Rio de Janeiro, with a treatment regimen based on Clarithromycin, Moxifloxacin, and Clofazimine. It is expected that her treatment will be completed by April 2024. The patient has opted not to undergone surgical removal of the nodules or abscesses.

### 2.2. Case 2

A 31-year-old Black woman, married, mother of one daughter, homemaker, reporting regular exercise with no history of previous illnesses or comorbidities, no reports of chronic medication or allergies, with vaccinations up to date, was admitted to the Infectious Diseases Service of HGR in April 2022, 47 days after undergoing plastic surgery comprising mastopexy, liposuction, and gluteal fat grafting. Her complaints included fever with chills, joint pain, redness, swelling, warmth, and significant purulent discharge from surgical wounds located in the gluteal and periumbilical regions. However, upon detailed discussions and a thorough review of her medical history, it emerged that approximately three months before the plastic surgery, she had received lipolytic enzyme application in her abdominal area at a private clinic in Boa Vista, Roraima, Brazil, performed by a nursing professional. Symptoms began on the 7th day post-operation (in February 2022). At that time, she consulted the operating surgeon, who prescribed Amoxicillin + Clavulanate for 10 days, but there was no improvement. Three days after completing the antibiotic course, on the twentieth post-operative day, she visited the emergency department at HGR.

Laboratory tests revealed leukocytosis (12,000 mm^3^) with a left shift (73% neutrophils), hemoglobin level of 11 g/dL, platelet count of 404,000 mm^3^, and an elevated inflammatory marker (CRP—C-reactive protein—84.720). Despite a ten-day treatment regimen involving Ciprofloxacin and Metronidazole, there was no observed improvement in her condition.

On the 30th post-operative day, she sought care from an infectious disease specialist due to the case’s complexity and unresponsive nature to antibiotic therapy. Consequently, she was admitted to *Hospital das Clínicas* (HC) and began treatment with Cefepime, later escalated to Meropenem and Teicoplanin. A non-contrast abdominal and pelvic Computed Tomography (CT) scan at admission to HC revealed irregular multifocal thickening/densification of the subcutaneous plane around the abdomen (mainly in the infraumbilical region) with adjacent fluid and reactive inguinal lymphadenopathies measuring up to 1.2 cm.

**Figure 2:**
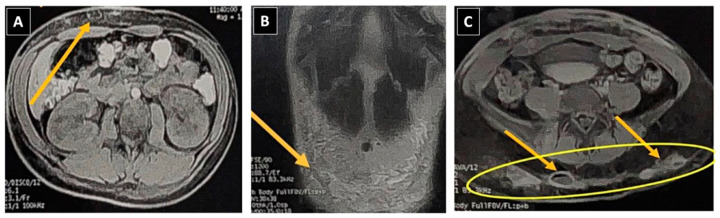
Magnetic Resonance Imaging (MRI) of the pelvic region. (**A**,**B**) Large collection in the hypogastrium measuring 12.3 × 2.0 cm, indicated by yellow arrows. (**C**) Abscesses in the gluteal regions, sizing at 3.5 × 2.0 cm on the right side and 4.2 × 2.4 cm on the left side, accompanied by a slight presence of free fluid within the pelvic cavity.

A guided puncture in the hypogastric region using ultrasound yielded purulent bloody fluid for microbiological analysis. She was then transferred back to the Infectious Diseases Service at HGR, maintaining a combined antibiotic regimen of Meropenem and Teicoplanin, with the addition of Amikacin. Upon admission, Nuclear Magnetic Resonance Imaging (MRI) of the pelvic and bilateral gluteal regions displayed postsurgical alterations in the subcutaneous tissue of the anterior abdominal wall in the hypogastrium and gluteal regions (Figure 2).

The patient underwent ultrasound-guided suprapubic and gluteal punctures; however, dense collections hindered complete drainage. This prompted evaluation by the General Surgery Service, which initiated drainage with a suprapubic drain maintained for 48 h. As the purulent fluid from the gluteal region was spontaneously draining, it was not intervened upon at that juncture. While hospitalized, the ongoing infection led to anemia due to increased consumption (hemoglobin of 9.8 mg/dL), necessitating treatment with Noripurum (iron hydroxide).

Considering the clinical response to antibiotic therapy, the absence of growth in blood and secretion cultures, and the persistent nature of lesions as observed in radiological assessments, secondary ultrasound-guided drainage was conducted on the suprapubic collection. Simultaneously, a sample of purulent secretion from a cutaneous fistula was obtained for microbiological identification. At this juncture, the initial microbiological result revealed the presence of Acid-Fast Bacilli (AFB 1+/4+).

Treatment commenced with a combination regimen including Clarithromycin, Rifampicin, Isoniazid, Pyrazinamide, and Ethambutol (RHZE) while awaiting culture and identification results. Subsequent mycobacterial analysis via the line probe assay (LPA) confirmed the strain as *Mycobacterium abscessus*, a member of the *Mycobacterium abscessus* complex. Sensitivity tests revealed resistance to Ciprofloxacin, Doxycycline, Moxifloxacin, and Clarithromycin, intermediate sensitivity to Cefoxitin, and susceptibility to Amikacin. Consequently, a combined therapy regimen was initiated, comprising Imipenem, Amikacin, Tigecycline, Linezolid, and Clarithromycin, targeting the initial phase to resolve abscesses. This was coupled with multiple surgical interventions to excise and manage infectious foci. Intravenous treatment spanned five months but was discontinued due to adverse reactions to Tigecycline. Subsequently, an additional two months of oral therapy involving Bactrim, Clofazimine, and Moxifloxacin was administered. Despite discontinuing treatment due to intolerance, she continues to exhibit a satisfactory response without any relapses.

### 2.3. Case 3

In August 2022, a 34-year-old Caucasian woman, a single mother and a nurse, who engaged in weightlifting six times per week, with no history of previous illnesses or comorbidities, no reports of chronic medication use or allergies, and presenting a complete vaccination history, visited an infectious disease specialist at HGR in Boa Vista, Roraima, Brazil, reporting skin lesions. She mentioned having undergone a lipolytic enzyme application in January 2022 to reduce localized fat. Without experiencing fever or pain, as a nurse, she recalled a conversation with another patient undergoing *Mycobacterium abscessus* treatment, prompting her evaluation. In their conversation, the other patient shared having received a similar enzyme treatment at the same clinic from the same professional. Subsequent to the August consultation, a sample was punctured, and nodules were excised for microbiological study and focus control. Given the strong epidemiological connection, the same empirical treatment regimen was initiated (Clarithromycin at 500 mg every 12 h, Moxifloxacin at 400 mg daily, and Clofazimine at 100 mg daily) until culture results were available. On 30 September 2022, positive culture results were obtained by using the line probe assay (LPA), identifying Mycobacterium *abscessus* subsp. *bolletii.* Sensitivity testing revealed susceptibility to Amikacin and resistance to Cefoxitin, Ciprofloxacin, Doxycycline, Tobramycin, Moxifloxacin, and Clarithromycin. Intravenous treatment was initiated, including Amikacin at 1 g daily for 15 days, followed by thrice-weekly doses, Ertapenem at 1 g/day, Linezolid at 600 mg every 12 h, and Tigecycline at 50 mg/day for 6 months (until March 2023). Concurrently, five additional nodules that appeared during this period despite combined antibiotic therapy were surgically excised. Tigecycline had to be discontinued due to side effects (nausea, general discomfort, and sinus tachycardia).

After the attack phase and surgical control of infection foci, oral therapy with Clofazimine, Clarithromycin, and Moxifloxacin at usual doses and Bactrim (Sulfamethoxazole/Trimethoprim) at 800/160 mg every 12 h was maintained. Treatment was paused for 30 days due to increased liver enzymes (AST, 68.86 U/L, and ALT, 129.94 U/L) and anemia (hemoglobin = 9.5 g/dL), despite daily folic acid use, with recovery upon its discontinuation.

**Figure 3:**
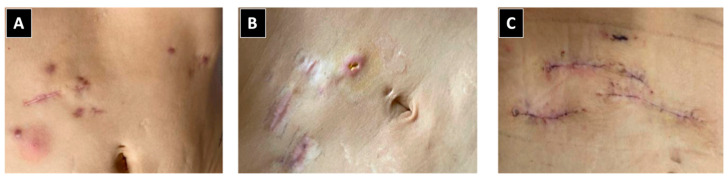
Progression of lesions caused by *Mycobacterium abscessus* after lipolytic enzyme application. (**A**) Nodular lesion featuring an erythematous base situated in the right upper quadrant of the abdomen. (**B**) Scars with subcutaneous nodules shown on ultrasound. (**C**) Scars post-surgical excision of the nodules.

After a year and a month of medication, a new surgical procedure was performed to remove residual nodules and fibrotic areas (Figure 3) identified via abdominal wall ultrasound, indicating the persistence of two adjacent residual subcutaneous nodules measuring 0.8 × 0.5 × 0.6 cm and 1.5 × 0.9 × 0.6 cm. They appeared hypoechoic with internal areas of higher echogenicity, regular contours, and no significant flow on Doppler, suggesting remaining infectious foci. Twenty days post-procedure and after 1 year and 2 months of treatment (still on Clofazimine, Moxifloxacin, and Clarithromycin), the patient remains well, without new abscesses, with treatment scheduled to conclude in January 2024.

## 3. Discussion

An outbreak is defined as any epidemic (i.e., a significant increase in cases of a specific disease) where cases are confined to a generally small and well-defined geographical area [15]. The three cases, despite their distinct complications and different timeframes for contamination, clinical manifestations, empirical treatment, and directed treatment, share something in common: long latency periods. In Case 2, the patient underwent enzyme application, and the infection remained latent for approximately 3 months. Only after a liposuction and fat grafting procedure did it manifest clinically, disseminating through the soft tissues where the suction cannula was used and infecting the grafted fat in the gluteal region, which served as a culture medium and proliferation site. The latency period was common to all three cases—2 months in case 1, 3 months in case 2, and 4 months in case 3—consistent with the literature that acknowledges this microorganism’s ability to remain asymptomatic within the host for long periods after contamination [10].

The second point of discussion is the symptomatic manifestations of non-tuberculous Mycobacteria (NTMs), as well as the challenge in suspecting this type of infection. As observed in the three cases, the clinical presentation exhibited by post-*M. abscessus* infection usually occurs between four and six weeks after the procedure. However, literature reports have noted manifestations months after contamination. The local picture typically includes nonspecific inflammatory signs like redness, induration, micro-abscesses, and serous drainage [6], evident in cases 1 and 3. Systemic symptoms like fever, chills, or other manifestations, commonly associated with infectious conditions, albeit rare, were observed in case 2. The surgery during the infection’s latent period disseminated it, justifying the patient’s extensive clinical presentation. According to the Brazilian recommendations for the diagnosis and treatment of diseases caused by non-tuberculous Mycobacteria, there are minimal reports on the non-pulmonary form of this condition [4], in line with the general literature, where the topic is scarcely covered.

Another significant challenge lies in the isolation and cultivation of this particular type of bacteria. In numerous healthcare facilities, routine cultures often prove ineffective in its identification. Furthermore, culture media like Lowenstein–Jensen, which are preferred for suspected Mycobacterium cases [16], are not consistently accessible. NTMs replicate more slowly in vitro compared with other bacterial species; consequently, these organisms might not be promptly identified in microbiological cultures, being frequently discarded after a standard incubation period, which does not allow for sufficient time for their growth.

The complexity of isolating *M. abscessus* in culture within the described cases—necessitating months for sample collection, obtaining positive results, and conducting sensitivity tests—coupled with the limited availability of optimal culture media, elucidates the diagnostic deadlock and the delayed onset of targeted treatment. Usually, these kind of skin lesions are treated with minor incisions, drainage, and empirical antibiotic therapy for common cutaneous germs (mostly oral cephalosporin) [6]. However, the patients did not respond positively, leading to a trial-and-error process and causing delays in the accurate diagnosis. Given the well-established epidemiological connections among the patients, the consistent subcutaneous enzyme application at the same clinic by the same professional, identical clinical presentations showing compatible signs and symptoms, and microbiological confirmation through culture and molecular biology of *M. abscessus*, no histopathological examination was required. Nevertheless, in the cases delineated in the article, we have a distinct outbreak, meticulously documented with epidemiological ties, robust clinical observations, and laboratory tests affirming the diagnosis (culture and molecular biology including species and subspecies identification), as well as an appropriate clinical response to tailored treatment [17].

This scenario exposes affected patients to prolonged use of empirical antibiotic therapy and derived side effects. In case 1, the difference in time between symptom onset and the final culture result with sensitivity testing was approximately 8 months, with the patient being exposed to four different antibiotics before starting targeted treatment. In case 2, the patient remained exposed to empirical antibiotic therapy for about 3 months, only obtaining the first positive culture result on the 57th day of hospitalization (the first sample was sent on 9 May 2022, with the result being released on 8 June 2022), needing to wait an additional 23 days for species identification and sensitivity testing (released on 1 July 2022). During this interval, she was exposed to over 10 antibiotics, most of which had no effect against *M. abscessus*. The patient in case 3, presenting similar symptoms to case 1 and having the same origin of the applied enzymes identified early, ended up receiving the correct treatment more quickly (even before the culture was released). However, this did not prevent the infection’s prolonged progression.

Macrolides are the only orally safe drugs considered effective in vitro against *M. abscessus* [6]. Thus, Clarithromycin was included in all treatment regimens from the beginning for the three patients. The recommended treatment duration is a minimum of four months, but this does not guarantee complete resolution of the infection, as observed in the management of the three patients. Treating them is, therefore, challenging due to the natural and acquired resistance of Mycobacteria to most currently available antibiotics [3].

The inappropriate use of sterilization techniques is believed to be largely responsible for these kinds of infections. Indeed, over the past decades, there has been an increase in cases of *M. abscessus* infections after liposuction in soft tissues [18], acupuncture [19], and mesotherapy [20]. Preventing these infections mainly involves advocating for good sterilization practices and hospital article processing [21]. In the case series presented, considering the epidemiological aspect that all patients underwent the aesthetic procedure in the same establishment and sought medical attention at the same service, it was possible to provide a faster diagnosis and treatment from the third case onwards, despite the unanimous delay in the availability of culture results. It is important to emphasize that the specifics of health surveillance procedures and the strategies implemented for investigating and controlling the outbreak were not covered in this study, as they are beyond its intended scope. However, we affirm that these measures were undertaken.

**Figure 4:**
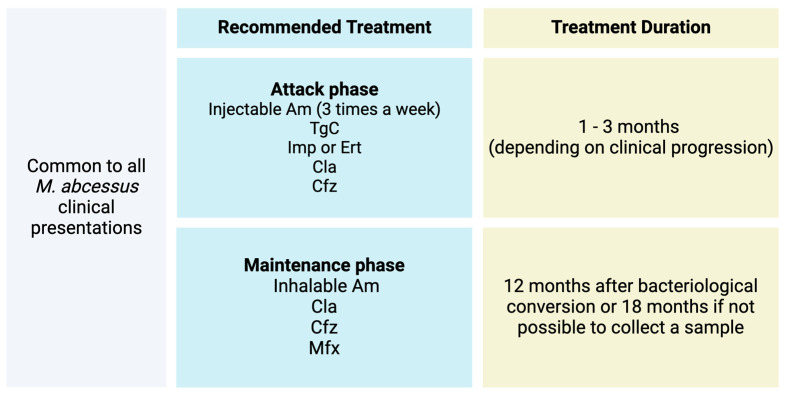
Recommended treatment for *Mycobacterium abscessus*. Subspcies: *M. abscessus* abscessus, *M. abscessus* massiliense, and *M. abscessus* bolletii. Modified from (4).

Despite the growing importance of the subject in public health matters, the number of published studies related to complications after minimally invasive aesthetic procedures, usually performed in Brazil by biomedical professionals, nurses, and pharmacists, among others specialized in aesthetics, is still limited [22]. There are predefined antibiotic regimens depending on the identified Mycobacterium species [4]. Concerning *M. abscessus*, the recommendations are displayed in Figure 4. However, the escalating complexity of resistance profiles poses challenges and complicates the straightforward resolution of *M. abscessus* infectious conditions, creating a sense of concern about this issue. Upon receipt of confirmatory microbiological tests for the three cases, they were treated with the therapeutic regimen recommended by the Ministry of Health (MS), with the exception that one patient did not receive carbapenems or glycylcyclines.

Intolerance to drug therapy also proved to be a barrier to treatment completion. In cases 2 and 3, during hospitalization, Tigecycline had to be suspended due to extreme general discomfort, nausea, and vomiting. Furthermore, in the latter case, there was even sinus tachycardia of up to 190 bpm—effects already described in the leaflet. After hospital discharge, the first patient also reported many adverse effects to oral medications, quinolone (Moxifloxacin) and macrolide (Clarithromycin), leading to non-adherence and loss of medication follow-up.

Another interesting point concerns the role of infectious focus control as part of the treatment and its level of importance. Indeed, patients subjected to focus control had shorter hospital stays and a 21% lower risk of mortality [23]. In cases 2 and 3, where focus control was performed (nodules removal and abscess drainage), the healing process was facilitated: in case 2, despite treatment abandonment, the patient remained without relapses, and in case 3, no new nodules were observed on ultrasound after the last removal, thus shortening the duration of antibiotic therapy. Aspiration, abscess drainage, or infected nodule excision proved effective even though none of these procedures occurred within the initial 12 h. However, in case 3, where no procedures were conducted, sporadic nodules still appeared, despite a good response to clinical treatment.

**Figure 5:**
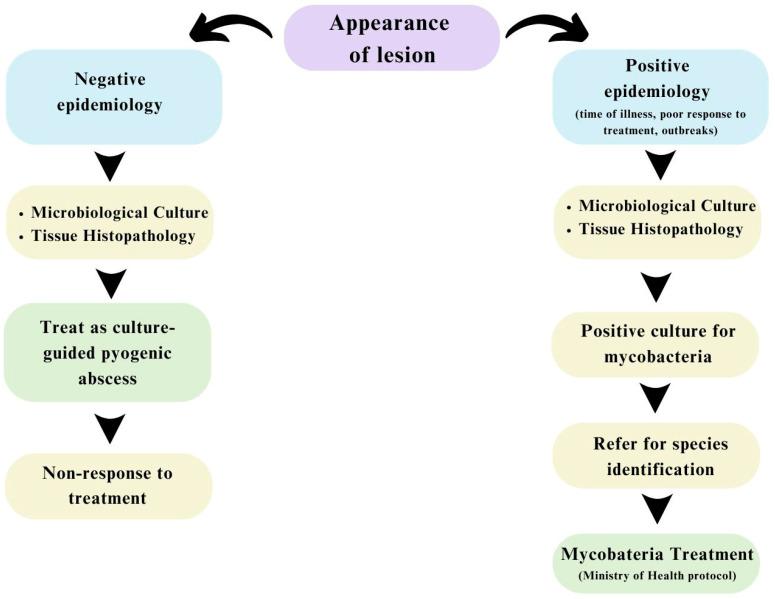
Flowchart for investigating non-pulmonary infections caused by non-tuberculous Mycobacteria.

Currently, the literature is lacking a comprehensive and explicit guideline for managing healing control in extrapulmonary mycobacterial infections. The Brazilian Ministry of Health exclusively focuses on monitoring pulmonary infections, utilizing X-rays, bacilloscopy, and serial assessments, omitting guidance on monitoring and healing control for conditions beyond the lungs. In all three cases, monitoring involved observing clinical signs and detecting nodules by using ultrasound. Yet, it is crucial to highlight the absence of a specific monitoring protocol for mycobacterial infections outside the pulmonary context. Given the diagnostic challenges and the complexity of the investigation, as well as the absence of specific protocols for non-pulmonary infections caused by non-tuberculous Mycobacteria, the local hospital and infectious disease physicians have been utilizing the following flowchart to assist in the investigation (Figure 5).

Considering the increasing significance of these infections, there is a pressing need to address this gap within the scientific community, fostering the development of an effective management framework for ensuring proper healing control. Furthermore, it is imperative to underscore that reporting cases of rapidly growing mycobacterial infections is mandatory. Healthcare professionals and institutions, whether public or private, must promptly report any suspected cases linked to healthcare procedures.

## 4. Conclusions

In light of the substantial presence of the beauty market in Brazil, paradoxically coupled with the limited discourse concerning the inherent risks and complications associated with aesthetic procedures, there emerges a clear imperative to elevate the discourse on this subject matter. Despite the considerable volume of beauty and aesthetic interventions, recorded incidents of infections resulting from these procedures remain notably sparse, not due to their absence but largely due to underreporting and the dearth of specific epidemiological investigations dedicated to such infections. Consequently, there exists a compelling necessity to broaden the scope of analysis and research concerning this domain to streamline diagnostics and fortify the management strategies for associated complications, thereby intensifying the fight against non-tuberculous Mycobacteria (NTM).

## Data Availability

Data available on request from the authors.
